# Setting goals with patients living with multimorbidity: qualitative analysis of general practice consultations

**DOI:** 10.3399/bjgp19X704129

**Published:** 2019-06-04

**Authors:** Charlotte Salter, Alice Shiner, Elizabeth Lenaghan, Jamie Murdoch, John A Ford, Sandra Winterburn, Nick Steel

**Affiliations:** Norwich Medical School, University of East Anglia, Norwich.; Norwich Medical School, University of East Anglia, Norwich.; Norwich Medical School, University of East Anglia, Norwich.; Norwich Medical School, University of East Anglia, Norwich.; Norwich Medical School, University of East Anglia, Norwich.; Norwich Medical School, University of East Anglia, Norwich.; Norwich Medical School, University of East Anglia, Norwich.

**Keywords:** doctor–patient communication, goal setting, goals, multimorbidity, patient care planning, patient-centred care, primary healthcare

## Abstract

**Background:**

Establishing patient goals is widely recommended as a way to deliver care that matters to the individual patient with multimorbidity, who may not be well served by single-disease guidelines. Though multimorbidity is now normal in general practice, little is known about how doctors and patients should set goals together.

**Aim:**

To determine the key components of the goal-setting process in general practice.

**Design and setting:**

In-depth qualitative analysis of goal-setting consultations in three UK general practices, as part of a larger feasibility trial. Focus groups with participating GPs and patients. The study took place between November 2016 and July 2018.

**Method:**

Activity analysis was applied to 10 hours of video-recorded doctor–patient interactions to explore key themes relating to how goal setting was attempted and achieved. Core challenges were identified and focus groups were analysed using thematic analysis.

**Results:**

A total of 22 patients and five GPs participated. Four main themes emerged around the goal-setting process: patient preparedness and engagement; eliciting and legitimising goals; collaborative action planning; and GP engagement. GPs were unanimously positive about their experience of goal setting and viewed it as a collaborative process. Patients liked having time to talk about what was most important to them. Challenges included eliciting goals from unprepared patients, and GPs taking control of the goal rather than working through it with the patient.

**Conclusion:**

Goal setting required time and energy from both parties. GPs had an important role in listening and bearing witness to their patients’ goals. Goal setting worked best when both GP and patient were prepared in advance.

## INTRODUCTION

Goal setting between physicians and patients can bring patient preferences to the centre of the consultation. Goal setting involves the sharing of realistic health and wellbeing goals, and is rooted in an understanding of patients’ priorities and preferences.^1^ It belongs in the long tradition in medicine of listening, understanding, and bearing witness,^2^ and in doing so represents one mechanism by which patient-centred communication might be enacted within consultations. Patient-centred communication has been advocated for decades^3^ and is a key tenet of the approach advocated for people with long-term conditions.^1^^,^^4^^,^^5^ Patients not only often want less medical intervention than their doctors think, but they also want continuity and access to a GP who knows about them as an individual.^6^ There are many factors preventing good communication in modern general practice,^7^^,^^8^ and remaining genuinely patient centred while exploring and negotiating patient priorities, options, and goals requires a high level of communicative competence.^9^

Goal setting may be particularly useful to facilitate patient-centred communication for patients with multimorbidity. However, despite four out of five consultations in general practice involving a patient with multimorbidity,^10^ such patients are often treated using single-disease, guideline-based management, which can increase treatment burden and harmful polypharmacy.^11^^,^^12^ Doctors and patients with multimorbidity working together on what really matters to patients can improve self-management,^5^ and may also increase GP job satisfaction. Further benefits may include reduced polypharmacy, hospital admissions, and costs through increased patient self-efficacy, improved treatment concordance, reduced adverse effects, and the encouragement of earlier appropriate contact with primary care.^13^^–^15

Despite National Institute for Health and Care Excellence recommendations,^1^ there is little published evidence about the process of setting goals for patients with multimorbidity in primary care. In this study the authors set out to answer the question ‘what are the key components of goal setting in general practice?’ through analysis of video recordings of patients and GPs during goal-setting consultations, within a trial to assess the feasibility of goal setting for patients with multimorbidity and at high risk of hospital admission.^16^

## METHOD

Data were collected during a cluster randomised controlled feasibility trial of goal setting compared with usual care planning in six general practices in the UK, as described elsewhere.^16^ In brief, patients who were within the top 2% at risk of unplanned admissions, eligible for a new care plan, living with ≥2 long-term conditions,^17^ able to communicate verbally, and deemed able to participate according to their GP were recruited. Three practices were randomised to a goal-setting intervention and three to a control group. The study took place between November 2016 and July 2018.

**How this fits in**

Setting goals with patients with multimorbidity in general practice is widely recommended, yet little is known about how it is done. The authors analysed 10 hours of video-recorded general practice consultations to determine the key elements of goal setting. These elements were: prior preparation for goal setting for patients and GPs, GPs legitimising or ‘bearing witness’ to the patients’ goals, and collaborative action planning. Findings suggest that goal setting can enhance collaboration, empower patients, and offer GPs personal satisfaction.

### Intervention

**Figure 1.**
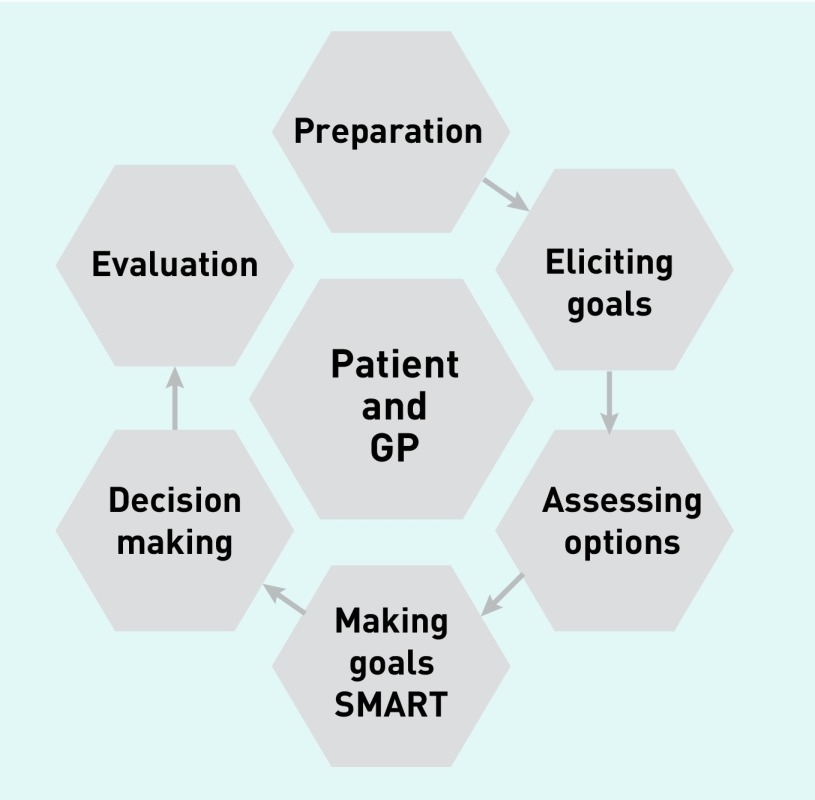
***Goal-setting training model.*** ***(SMART = Specific, Measurable, Attainable, Realistic/Relevant, and Time bound).***

Building on established models of communication and shared decision making,^18^^–^20 the authors developed a working training model that adopted a structured, patient-centred stepped approach. Steps included preparation; goal elicitation; assessing options; making goals SMART (Specific, Measurable, Attainable, Realistic/Relevant, and Time bound); decision making; and evaluation (Figure 1 shows an infographic of these steps; see also Box 1). GPs from the three intervention practices participated in a 3-hour experiential workshop, including a discussion of key principles, skill spotting, using video examples of goal setting in action, and role-play.

**Box 1.** Analytical framework for analysing videos

**Emergent theme (mechanism)**	**Gross structure informed by model of shared decision making^19^^,^^20^**	**Evidence of activity and interactional competencies (skills used — not mutually exclusive)**
Patient preparation and engagement	Pre-beginning Preparation and opening	GP checks patient understanding of goal setting Patient attends with completed paperwork or refers to it Patient indicates they have discussed goals, for example, with family, friends Both parties establish the agenda GP invites patient to lead Patient initiates discussion about own goals/priorities
Eliciting and legitimising goals	Eliciting goals Assessing options	GP encourages early identification of patient priority/priorities GP listens attentively without interruption GP supports and validates patient’s view about importance of goal GP picks up and explores cues and clues Patient talks openly about what is important to them and why GP explores patient’s personal circumstances GP explores patient goal(s) GP and patient discuss options for goals
Collaborative action planning	Making goals SMART Decision making Summary Evaluation Closure	GP invests time in this process GP and patient deliberate together Patient describes own thinking on steps to take to achieve goal Choices discussed by both parties to explore and set goals Pros and cons of options are discussed A SMART goal informed action plan is negotiated GP or patient record agreed goal and current level of attainment

SMART = Specific, Measurable, Attainable, Realistic/Relevant, and Time bound.

Participating patients were provided with a three-page A4 goal-setting sheet (GSS) with three trigger questions and room for note making. Patients were invited to attend two appointments with a participating GP: a 20-minute goal-setting consultation to discuss and record their goals on a form, and a 10-minute follow-up 6 months later. The GSS and goal-recording forms, for both consultations, are available from the authors on request. Consultations were video-recorded and transcribed verbatim, including non-verbal interactions, based on principles from Jefferson’s transcription conventions.^21^

Two focus groups were held with participants, one with GPs and one with patients, serving as a form of triangulation with findings from the video analysis to assess the acceptability of the goal-setting process and explore emergent themes. One-to-one interviews were undertaken with patient or GP participants who wanted to contribute but could not attend the focus group. Focus groups were guided by a topic guide that was informed by initial analysis of the consultations, audio-recorded, and transcribed.

### Data analysis

Goal-setting consultation recordings were watched/listened to individually by the research analysis team (two social scientists with expertise in communication skills, a GP, and a study researcher), with the team meeting weekly for 6 months to discuss and reflect on the emerging results. Using an approach the authors have previously applied to the analysis of provider–patient consultations, transcripts were analysed by first describing the gross consultation structure and delineating activity types.^22^ The gross structure comprised nine steps/activities based on the original training model: pre-beginning, preparation and opening, eliciting goals, assessing options, making goals SMART, decision making, summary, evaluation, and closure. A subsequent activity-based analysis examined verbal and non-verbal evidence from the consultations to explore how these key aspects of the goal-setting consultation were enacted by GPs and patients. This involved coding of a priori tasks and their related competencies (skills) in core aspects of patient-centred communication (Box 1). A central focus was on patterns of interaction within activities,^23^ themes relating to how goal setting was attempted and achieved, and the management of communication challenges.^24^ Open coding was used for emergent findings. Focus group data were indexed and charted by four of the researchers using thematic analysis to generate codes and reveal themes,^25^ building on the findings of the activity-based analysis.

The authors actively sought dissenting cases to fully explore the complexity of goal setting and its communicative challenges. They also sought to validate their initial findings through sharing a sub-sample of four transcripts with two public and patient involvement (PPI) representatives with expertise in the formal and informal care sector. This served as a form of member checking and helped to strengthen the validity for the analysis. Four PPI representatives contributed to a consultation event to discuss findings.

This article reports on the key themes identified throughout the goal-setting process using extracts from the goal-setting consultations and focus group interviews.

## RESULTS

Four male GPs and one female GP from three market-town practices took part in the intervention (Table 1). Of the 24 patients enrolled from the goal-setting practices (Table 2), 22 patients completed the initial consultations, and 18 attended follow-up consultations, amounting to 673 minutes of recorded consultations. Most patients set two or three goals, all related to health and wellbeing. The nature of the goals set is described elsewhere.^16^

**Table 1.** Characteristics of practices randomised to goal-setting intervention, and of participating GPs in those practices

**Practice characteristics**	**Practice 1**	**Practice 2**	**Practice 3**
Practice rurality^a^	Village — less sparse	Town and fringe — sparse	Town and fringe — sparse
Practice population, range, *n*	5000 to 9900	10 000 to 14 900	5000 to 9900
IMD decile	7	5	7

**Characteristics of participating GPs**			
Male sex, *n*	2	1	1
Female sex, *n*	0	1	0
Employment status	Partners, 2 PT	Partners, 2 FT	Partner, PT
Time qualified, years	GP014, >20; GP018, 10 to 20	GP025, <10; GP026, 10 to 20	GP038, 10 to 20

a *Office for National Statistics indicator 2011.^26^* *FT = full time. IMD = Index of Multiple Deprivation (1 = most deprived and 10 = least deprived). Partner = GP with responsibility for the practice. PT = part time.*

**Table 2.** Baseline characteristics of patients enrolled in goal-setting group, *N* = 24

**Characteristics**	**Value**
Female, *n* (%)	13 (54)
Age, years, mean (SD)	80 (9)
Patients who saw their usual GP^a^ for goal-setting consultations, *n* (%)	5 (21)
Number of medications, median (IQR)	13 (10 to 17)
Number of diagnoses,^b^ median (IQR)	5 (3 to 6)

a Usual GP as defined by patient on enrolment to study.

b *Based on Barnett list.^17^* *IQR = interquartile range. SD = standard deviation.*

Four GPs attended a focus group lasting 100 minutes, and the fifth GP was interviewed one-to-one (25 minutes). All 22 patients were invited to attend a focus group, except two, who declined to be approached, and one deceased patient. Of the remaining 19, eleven expressed an interest, but only six were able to attend on the day. Three carers were present at the focus group, which lasted 12 minutes. Two patients took part in a telephone interview (lasting 33 and 23 minutes).

**Figure 2.**
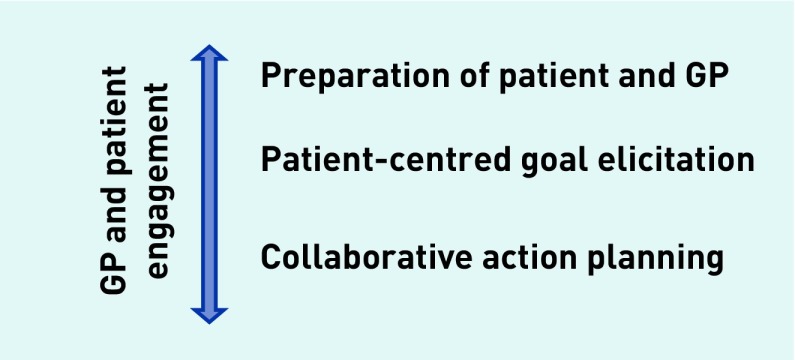
***A model for effective goal setting.***

Four main themes emerged concerning components of the goal-setting process required to make it effective and included: patient preparedness and engagement; eliciting and legitimising goals; collaborative action planning; and GP engagement. This can be represented as a model (Figure 2).

### Patient preparedness and engagement

The extent to which patients were prepared for the consultation had an impact on whether consultations displayed evidence of patient-led interactions. Preparation included patients discussing goals with family or carers and completing the GSS. Box 2 shows an example of how being prepared and committed to the process allowed Patient (Pt)109 (male [M], aged 70–74 years) to disclose their goals to the GP within the opening moments. The patient then went on to discuss the three personal goals they had in mind.

**Box 2.** Example of patient preparation^a^

**Time, minutes:seconds**	**Line**	**Speaker**	**Excerpt**
00:03	1	GP018:	I’m er Dr GP018 (0.5) take a seat.
	**2**	**Pt109:**	**Thank you sir (1.9)**
	3	GP018:	Right, yeah so hh=
	**4** (6 lines omitted)	**Pt109:**	**=I filled in paperwork, as instructed by the lady.**

00:20	11	GP018:	Do you feel you had a (0.3) chance to, sort of, ask a lot questions and get a good understanding of what this is [all about, yeah?]
	12		

**00:25**	**13**	**Pt109:**	**[Yes I did yes yes I did] (0.3)**

00:28	14	GP018:	And how did you find, um (0.5), er setting goals or having a think about what sort of things you were? (0.7)
	15		
	**16**	**Pt109:**	**Well, there were several things I thought of that I’d like to do right**
	17	GP018:	Mmm=

**00:40**	**18**	**Pt109:**	**Um****, (1.3) hhh I take quite a high dose of (0.5) different types of tablets=**
	19	GP018:	=Yeah, yeah.
	**20**	**Pt109:**	**And I’d like to er see if there’s any possible way to cut some back=**
	21	GP018:	=Okay, yeah.
	**22** (10 lines omitted)	**Pt109:**	**Right, (2.0) and I walk to a, roughly about a mile a day, right?**

01:19	33	GP018:	Okay (0.2) yes, so, um, yeah, we’ll definitely go through those — sounds like you’ve had a good — good think about it, actually, and come up with some good stuff.
	34		

a [Number] indicates a pause measured in seconds inside brackets. [ ] marks start and end of overlapping talk. Underlining locates emphasis. Equal sign indicates no gap between two lines of talk. Pt = patient.

When asked at the end of the consultation if the patient had any questions, Pt109 stated that they were able to discuss what they were interested in and have questions answered. Pt209 (Female [F], 90–94 years), who had written extensively about their goals on the GSS, also described a personalised understanding of setting goals: *‘… well it is something I particularly want to do’*.

At the follow-up consultation 6 months later Pt109 had partially attained all their goals, according to the goal attainment scoring method used, despite further complex health issues arising. The patient stated they were *‘quite pleased’* and thought they had got on *‘very well’* with the goals and *‘felt better’* in themselves. They also stated in the focus group that they:

*‘*… *wouldn’t have done it without the* [goal] *plan because I don’t think I’d have had the willpower to manufacture what I was doing.’*

(Pt109, M, 70–74 years)

Where a patient had not prepared for the consultation, invariably the GP took more control. Conversely, GPs perceived they had to take less control when a patient came prepared and engaged with the process:

‘Those who came in with really clear idea, goals, they’ve done their homework, they knew what it was all about and then you had those who came in who were just like, oh no I haven’t done that, no you tell me doctor, what shall I do, what, what shall my goals be and it is like.’

(Focus Group [FG], GP018)

Several patients who were also carers had not arrived with goals in mind, one even stating they were doing it because *‘it’s just to help old people isn’t it?’* (Pt201 F, 75–79 years, GP025). When patients had not prepared, there were no examples of the goal-setting process going well. There were examples of patients being prepared but still the intervention was problematic, but for other reasons.

Sometimes patients simply seemed to need reassurance that the GP really did want them to prioritise their own goals.

### Eliciting and legitimising goals

Goal elicitation was a shared process that involved relationship building between patient and GP. It required more than simply identifying or listing ambitions. Patients appeared able to bring goals they might not otherwise have brought to the consultation as health issues, and GPs often acted as catalysts to the goal-setting process. In the extract shown in Box 3, Pt304 (F, 65–69 years) prioritises their mental health, saying at both the initial and follow-up consultation that this was not something they had previously felt able to *‘bring to a GP’*.

**Box 3.** Example of eliciting and legitimising goals^a^

**Time, minutes:seconds**	**Line**	**Speaker**	**Excerpt**
00:31	1	GP038:	Okay. What else matters to you at the moment, apart from that?

**01:21**	**2**	**Pt304:**	**I, I’m, well, I’ve had this small heart attack and, of course er I’m always anxious that um (0.5) there will be a repeat, or a stroke or something=**
	**3**		
	4	GP038:	=Okay
	**5**	**Pt304:**	**But and I’d like to (0.3) somehow not be so worried about that as a future and also, I realise I suffer quite badly from anxiety and sometimes occasional depression and I think the anxiety sort of makes everything worse=**
	**6**		
	**7**		
	8	GP038:	=Okay=

**01:42**	**9**	**Pt304:**	**=And probably impacts on my (0.2) health yeah=**
	10	GP038:	=Okay=
	**11**	**Pt304:**	**And certainly my blood pressure.**
	12	GP038:	Okay

a (Number) indicates a pause measured in tenths of a second inside brackets. [ ] marks start and end of overlapping talk. Underlining locates emphasis. Equal sign indicates no gap between two lines of talk. Pt = patient.

This example is one of a number that illustrate how the process, including validation from the GP, could act as legitimising the patients’ priorities and goals. Recognition of this role was also noted by GPs:

‘*I think that whole thing, I think what’s coming from the families as well, you know, by you as a GP interviewing them and setting targets, are pretty much authorising to do something he wants to do and that’s empowering them as well.’*

(Interview, GP038)

Legitimation was often tacit. In Box 4 Pt205 (M, 90–94 years) had set a goal to *‘meet a new partner’* as the patient was very lonely since their wife died. The GP acted as a catalyst by validating the patient’s goals (lines 25–27) and offering advice about the next step they could take (lines 117–119). Having been hesitant to raise it initially, Pt205 was visibly delighted when they returned at 6 months, confirming they had been to the group recommended by the GP and other groups. Most importantly the patient achieved their goal by meeting *‘a lady* [and] *that breaks up the loneliness* [and] *is brilliant’*. At the patient focus group the patient was pleased with achieving their goal, describing their GP as *‘brilliant’* though they did not know exactly what it was in the process that enabled it:

‘I’m going to be quite honest, we had the conversation and I was saying and I kept, I, I kept puzzled in my mind what or how is this going to help for me to achieve and I kept thinking why is she asking … even now I still can’t see how this has done that for me to achieve what I want to achieve.’

(Pt205, M, 90–94 years)

**Box 4.** Example of collaborative action planning^a^

**Time, minutes:seconds**	**Line**	**Speaker**	**Excerpt**
06:09	1	GP025:	No, oh well we’ve got a few nice things to work on here (0.2). Sounds like meeting people of a similar age is the one thing that’s really [in your]
	2		
	**3**	**Pt205:**	**[I do]**
	4	GP025:	[Big goal]

06:20	**5**	**Pt205:**	**[That is something] I want to do.**
	19 lines omitted		

07:07	25	GP025:	No, well let’s move on to so I think that we’ve let’s write those down because I think they’re excellent, I think you’ve come up with some really good ones there (0.6). So um the first one is meet people (2.9) (GP025 writes on sheet)
	26		
	27		
	4 lines omitted		
	**32**	**Pt205:**	**I do go to the one at the church erm (2.0) bereavement thing, I go there**
	33	GP025:	[Ah]

07:35	**34**	**Pt205:**	**[So I meet people there]**
	75 lines omitted		

10:31	110	GP025:	Yeah (0.3), yeah, so we’re looking for groups where you’ll meet other [females by the sounds of it]
	111		
	**112**	**Pt205:**	**[An’ that’s right that’s it]**
	113	GP025:	By
	**114**	**Pt205:**	**[Just]**
	**115**	**GP025:**	**[Yeah]**
	**116**	**Pt205:**	**er, go out, have a cup of tea, have a walk an’ [other things]**
	117	GP025:	[Yeah] yeah,
	118		have you been to the um (0.2) (church) group of (0.4) which is um (0.2) in (town). I got their leaflet somewhere? (0.9) Let me have a look (0.7) here we are.
	119		

a [Number] indicates a pause measured in seconds inside brackets. [ ] marks start and end of overlapping talk. Underlining locates emphasis. Equal sign indicates no gap between two lines of talk. Pt = patient.

For many patients the GPs’ role had been a form of ‘moral support’ and seemed to be to listen, validate, and support patients to articulate their goals rather than take action. For Pt105 (M, 75–79 years) this involved a discussion about how he wanted to inform his large extended family that he was dying.

Bearing witness and validating patients’ priorities highlight the power of the doctor to explore, legitimise, and enable patient priorities and goals:

‘Yes recognising the therapeutic powers of a consultation, the longer the consultation, the more people feel listened to, the more therapeutic.’

(FG, GP026)

Patients seemed to have gained something unquantifiable from the consultation: Pt105 talked about the experience (of talking) giving him *‘insight’*, and Pt205 could not identify what happened, but something acted as a catalyst. GPs in the focus group reflected, without prompt, that they were conscious of the therapeutic nature of their role.

GP018 contemplated both their role as clinician and positive counsel:

‘*I suppose that’s what motivator type* [sic] *as well, you know, um and positive counsel really um, I think there was a gentleman with quite late-stage lung disease who wanted his breathing to be good enough that he could go fishing with his son … and we did everything we could but at 6 months he couldn’t and perhaps he got a little bit worse but in many ways it actually gave us a real kind of um forum where he would really sort of come to terms with a lot of the palliative side of his condition and he put a lot of his affairs into order and he didn’t feel bad at all about not meeting his goals when it came to feeling less breathless and in many ways he was accepting of that part of his, you know, terminal.*’

(GP018)

Though no goal raised by a patient was off limits, GPs did confess to finding some goals more challenging, especially where they lacked knowledge or information about services. In addition, some goals were missed or not legitimised by GPs or actively contested. An example was when a patient specifically raised the goal of managing their diabetes without medication. Rather than explore this the GP appeared to ignore the patient’s concern making the decision that they were:

‘a patient that should be on medication for diabetes.’

(GP026; Pt202, M, 80–84 years).

### Collaborative action planning

One of the most important aspects of goal setting was the ability of the patient and the GP to discuss, formulate, and agree the goal specifics. This process involved a significant investment of time, negotiation, deliberation, and shared decision making about the steps towards goal attainment, as well as setting a nominal target. However, making goals measurable could overcomplicate and distance the patient from their own goal, and sometimes it became unrealistic to reach a goal within the timeframe, for example, due to unanticipated ill health. Yet still the process could be helpful for both; and in assessing a goal the skill was in knowing if a goal had been achieved or felt successful to a patient, rather than the GP forcing a target.

There were times when GPs felt the process involved *‘unknown territory’* (GP014) and, interestingly, GPs in the focus group described their role as ‘collaborative’ and as a ‘facilitator’ of the goal-setting process:

‘… so, yes just to facilitate them assessing their own goals um and then holding them to them, so um, so it’s, yeah basically that’s how I saw myself, you know. The initial meeting was to facilitate setting them and then 6-month review to try and hold up a mirror and see what we’d achieved and, and then try and set some new ones.’

(GP026)

‘Yeah I would agree that you felt the facilitator, an interesting thing was actually how you then go about it.’

(FG, GP018)

Highlighting the fact that it was about setting up a process, a patient at the focus group shared:

*‘*… *losing weight is an ongoing process, you know it’s not like oh yes in 6 months you will, it’s just something* [goal setting] *that helps me to focus on things that are important.’*

(FG, Pt111, F, 55–59 years)

The process was supported by patients having continuity in knowing they would see the same doctor again; as Pt109 summed it up: *‘*[When] *you see a different person nothing seems to follow up.’*

Box 5 shows a clear example of this collaborative process taking place within a goal-setting consultation. Pt206 (F, 70–74 years) responded in the opening minutes of the consultation to the GP’s suggestion: *‘if there’s anything* (that) *immediately jumps out at you as something you’d like to try and achieve’* with a definitive *‘I’d like to put on more weight’*. After several minutes of exploration of this priority and discussion about appetite loss and digestive problems, GP026 clarified the priority and explored exactly how the goal could be specified, actioned, and ultimately measured; the GP and patient collaborated and negotiated to agree a realistic target (see lines 14–28).

**Box 5.** Example of collaborative action planning^a^

**Time, minutes:seconds**	**Line**	**Speaker**	**Excerpt**
13:01	1	GP026	So it’s about eating er so what I need to do is put, so we need to write down here our goal=
	2		
	**3**	**Pt206**	**=Right**
	4 lines omitted		

13:19	8	GP026	So — (0.5) goal (0.2) to gain weight.
	**9**	**Pt206**	**Please, yes.**
	1 line omitted		
	11	GP026	Okay (.) Now — (0.5) we need to make that goal (0.9) in a way that we can think about whether we’ve achieved anything in 6 months’ time.
	12		
	**13**	**Pt206**	**Right=**
	14	GP026	=Erm, and so we want to put some numbers on it to make it specific. You’re 33 kilos now (0.7)
	15		
	**16**	**Pt206**	**Yeah**
	17	GP026	It would be very ambitious I think in 6 months to put [10 per cent weight]
	**18**	**Pt026**	[yeah]
	19	GP026	on which would be another 3 kilos, half a stone that would be very [ambitious]
	20		
	**21**	**Pt206**	**[Won’t] yeah but it would be lovely wouldn’t it**
	22	GP026	It would now we can be ambitious (0.2) but we want to try and achieve it=
	**23**	**Pt206**	**=Yeah I think to be that ambitious (1.5) I would probably do something silly and make myself violently sick.**
	**24**		
	25	GP026	So we don’t [want to do that]
	**26**	**Pt206**	**[So we don’t want] that, no**
	27	GP026	So I think we ought to try and tailor, tailor it down a bit=

**14:12**	**28**	**Pt206**	**=Yes** **cer****tainly**

a [Number] indicates a pause measured in seconds inside brackets. (.) indicates a pause of less than 0.2 tenths of a second. [ ] marks start and end of overlapping talk. Underlining locates emphasis. Equal sign indicates no gap between two lines of talk. Pt = patient.

The GP’s role was clear: prescribe high-energy drinks and refer to a dietician. The patient, however, owned and was clearly motivated by the goal, having stated that they were keen to get stronger and lessen their falls and fracture risk. This had been an ongoing health issue but it appeared to be the first time the patient had decided to prioritise it. What was key to this goal was not only that it was the patient’s priority, turned into a realistic goal, but also that the goal itself is collaboratively agreed and actioned. This patient gained >2 kg of the target at the 6-month follow-up consultation.

### GP engagement

Just as patient buy-in and engagement was essential, so was GP engagement. GPs valued the process and were unanimous in their enthusiasm for setting goals with patients. Several reported they had already started to use the core principles with other patients including the idea of asking the patient what really mattered to them most:

‘It kind of made you appreciate how much good, good medicine, good patient–doctor interaction you can get in that period and how useful it can be and how, how nice it is to get to know your patients that little bit more, yeah.’

(GP018)

‘Yes, I found it actually, it was almost a pleasure to see you had one of the patients coming up … um rather than dealing with today, well firefighting today’s problem, so, I enjoyed that aspect of it.’

(FG, GP026)

Though the process and procedures (including paperwork) were seen as useful, GPs described personal satisfaction with the consultations regardless of the type of goal set and/or attained, highlighting its therapeutic power. They valued that goals were patient centred and focused on things that could change. Furthermore, it appeared to give a patient permission to focus on a particular priority and GPs the opportunity to get to know their patients better and explore problems more fully. It was the opposite of box ticking and GPs were keen to stress that goals should not become tests or provoke anxiety; only one goal (or even no goals) was fine:

‘The joy of it, you know what, the joy, the joy of it was not having to tick box really much at all and having stuff written down, pen and paper.’

(GP018)

‘Um but I, I think it has had some subtle effect in terms of listening and waiting “what do you want” or “what would be an achievement” and the, you know, another example is the patient who, whose goal was to walk around the block again um, he didn’t achieve it because he didn’t, you couldn’t initiate any action to do it but that clarified so much for him and if that’s the goal, then it doesn’t matter whether that’s with or without heart failure, for example, and I find that a really good benefit of whatever intervention measured by a real-life goal is much easier to sell than the benefit of lowering cholesterol by X per cent.’

(FG, GP014)

Although GPs reported that the skills they used were not new in terms of communicating across complex situations and patient problems, they did report an increase in confidence following the training and appreciated the *‘step-by-step’* (GP014) approach.

## DISCUSSION

### Summary

The GPs had an important role in listening and bearing witness to their patients’ goals, even when it was not always clear how or whether the goal would be achieved. This seemed to work better when the GP and patient were prepared to set goals and for a different, more equal, and balanced consultation. The three main components that made goal setting effective were patient and GP engagement and preparation, supportive goal elicitation, and collaborative action planning (Figure 2). Patients liked having time to discuss what was most important to them about their health. They liked knowing they would see the same GP, as personal continuity of care was rare in usual practice and particularly valued for goal setting. GPs valued the process and time to deliver person-centred care.

Goal setting required time and energy by GP and patient, and there were challenges. For example, some patients were unclear what the consultation and research were about, and had not prepared goals; some GPs struggled to identify suitable goals that they could assist with; other GPs wrestled with which goals should be included. There were problems with making some goals measurable and achievable, either because the goal did not lend itself to being measured, or because the GP’s attempt to configure an activity into a formal goal with a pre-specified time point overcomplicated and distanced the patient from their own goal.

### Strengths and limitations

This research is the first to focus in-depth on doctor–patient communication during goal-setting consultations in primary care. Video analysis of more than 10 hours of goal-setting consultations enabled significant learning about healthcare communication, with transparency from detailed transcriptions and video analysis, and the involvement of an interdisciplinary study team.

Patients who participated were fairly representative of people living with ≥2 long-term conditions and among the most at risk of unplanned hospital admissions. Without exception they were living with declining health and life-limiting conditions. Focus groups enabled rigour and data triangulation of analytical interpretations of video data, as did the PPI representative involvement in the analysis. Limitations include that the GPs and patients had self-selected to take part in the feasibility study, though were randomised to the intervention arm.

This feasibility study took place in only three intervention practices in the East of England involving five GPs and 22 patients. It is known that even where practitioners set out to discover the patient’s own personal goals, long established patterns of interaction and role relationship by both parties can mean doctors assume and maintain power and patients passively or actively resist. However, findings presented here provide important insights into the circumstances under which goal setting can be beneficial for both GP and patient, providing theoretical propositions for testing in a larger definitive trial.

### Comparison with existing literature

There are few other comparable studies. A recent systematic review of goal setting with patients who were older confirmed the need for studies to examine the effects of personalised care planning on goal attainment, *‘especially patient’s personal goals as opposed to goals determined by clinicians or researchers’*.^27^ Kangovi *et al* found that patients with chronic conditions from low socioeconomic groups thought broadly about their goals when encouraged to.^28^ Most published literature on goal setting suggests goals are often constrained or amended by the healthcare team, frequently clinically prioritised rather than patient prioritised, amended or constrained by the clinician, and reoriented to a biomedical focus,^29^^–^31 and, in rehabilitation settings, there is evidence that patients may be ill prepared to play a more proactive role and therefore not have goals in mind.^32^

### Implications for research and practice

Further analysis of this body of data will investigate the detailed interactional negotiation of goal setting, and additional research is needed to explore the impact of lower health literacy and socioeconomic and mental health status on goal setting for patients with multimorbidity. Nonetheless, this study has managed to offer useful evidence for an approach that GPs can use to work effectively with patients who have multiple long-term conditions.

There is need for such an approach: GP workload is increasing, in part due to the demands of the growing population with multimorbidity. The approach offered in this article was acceptable to and valued by GPs. Furthermore, it may be easily translated into practice as the steps involved (GP and patient preparation, patient-centred goal elicitation, and collaborative action planning) correlate with existing models of communication skills for primary care.

## References

[1] National Institute for Health and Care Excellence (2016). Multimorbidity: clinical assessment and management NG56.

[2] Heath I (2012). The art of doing nothing.. Eur J Gen Pract.

[3] McWhinney IR (1993). Why we need a new clinical method. Scand J Prim Health Care.

[4] Department of Health (2014). Transforming primary care: safe, proactive, personalised care for those who need it most.

[5] Coulter A, Entwistle V, Eccles A (2015). Personalised care planning for adults with chronic or long-term health conditions (Review).. Cochrane Database Syst Rev.

[6] Levene LS, Baker R, Walker N (2018). Predicting declines in perceived relationship continuity using practice deprivation scores: a longitudinal study in primary care.. Br J Gen Pract.

[7] Launer J (2017). Is there a crisis in clinical consultations?. Postgrad Med J.

[8] Bower P, Reeves D, Roland M (2013). Care planning in the treatment of long term conditions — final report of the CAPITOL project.

[9] Parry RH (2004). Communication during goal setting in physiotherapy treatment session. Clin Rehabil.

[10] Salisbury C, Johnson L, Purdy S (2011). Epidemiology and impact of multimorbidity in primary care: a retrospective cohort study.. Br J Gen Pract.

[11] Wilkie P (2015). Really putting patients first: ensuring significant involvement for patients in healthcare decision making.. Br J Gen Pract.

[12] Mercer SW, Fitzpatrick B, Guthrie B (2016). The CARE Plus study — a whole-system intervention to improve quality of life of primary care patients with multimorbidity in areas of high socioeconomic deprivation: exploratory cluster randomised controlled trial and cost-utility analysis. BMC Med.

[13] Coulter A, Roberts S, Dixon A (2013). Delivering better services for people with long-term conditions. Building the house of care.

[14] Mulley AG, Trimble C, Elwyn G (2012). Stop the silent misdiagnosis: patients’ preferences matter. BMJ.

[15] Steel N (2000). Thresholds for taking antihypertensive drugs in different professional and lay groups: questionnaire survey.. BMJ.

[16] Ford JA, Lenaghan E, Salter C Can goal-setting for patients with multimorbidity improve outcomes in primary care? Cluster randomised feasibility trial.. BMJ Open.

[17] Barnett K, Mercer SW, Norbury M (2012). Epidemiology of multimorbidity and implications for health care, research, and medical education: a cross-sectional study. Lancet.

[18] Silverman J, Kurtz S, Draper J (2005). Skills for communicating with patients.

[19] Elwyn G, Frosch D, Thomson R (2012). Shared decision making: a model for clinical practice. J Gen Intern Med.

[20] Elwyn G, Durand MA, Song J (2017). A three-talk model for shared decision making: multistage consultation process.. BMJ.

[21] Jefferson G, Lerner G (2004). Glossary of transcript symbols with an introduction. Conversation analysis: studies from the first generation.

[22] Murdoch J, Barnes R, Pooler J (2015). The impact of using computer decision-support software in primary care nurse-led telephone triage: interactional dilemmas and conversational consequences. Soc Sci Med.

[23] Rampton B (2014). Gumperz and governmentality in the 21st century: interaction, power and subjectivity..

[24] Parry RH, Land V (2013). Systematically reviewing and synthesizing evidence from conversation analytic and related discursive research to inform healthcare communication practice and policy: an illustrated guide. BMC Med Res Methodol.

[25] Braun V, Clarke V (2006). Using thematic analysis in psychology. Qual Res Psychol.

[26] Office for National Statistics (2011). 2011 Rural/urban classification.. http://www.ons.gov.uk/ons/guide-method/geography/products/area-classifications/2011-rural-urban/index.html.

[27] Vermunt NPCA, Harmsen M, Westert GP (2017). Collaborative goal setting with elderly patients with chronic disease or multimorbidity: a systematic review.. BMC Geriatr.

[28] Kangovi S, Mitra N, Smith RA (2017). Decision-making and goal-setting in chronic disease management: baseline findings of a randomized controlled trial. Patient Educ Couns.

[29] Corser W, Holmes-Rovner M, Lein C, Gossain V (2007). A shared decision-making primary care intervention for type 2 diabetes. Diabetes Educ.

[30] Yu CH, Stacey D, Sale J (2014). Designing and evaluating an interprofessional shared decision-making and goal-setting decision aid for patients with diabetes in clinical care — systematic decision aid development and study protocol.. Implement Sci.

[31] Hoskins G, Abhyankar P, Taylor AD (2013). Goal-setting intervention in patients with active asthma: protocol for a pilot cluster-randomised controlled trial. Trials.

[32] Schoeb V, Staffoni L, Parry R, Pilnick A (2014). ‘What do you expect from physiotherapy?’: a detailed analysis of goal setting in physiotherapy. Disabil Rehabil.

